# Supplementary material for: Exploring the behavioral reactions to a mirror in the nocturnal grey mouse lemur: sex differences in avoidance
Source: PeerJ. 2021 May 13;9:e11393. doi: 10.7717/peerj.11393 (PMC8126259; doi:10.7717/peerj.11393)

**Supplements: results for session 2**

The repeated measures ANOVA on the first principal component (PC1) score for the session 2 revealed no effect of sex (P > 0.42, df=1, F = 0.63) or context (P > 0.99, df=1.7, F = 0.0003), and a significant interaction between the two factors (P = 0.026, df=1.7, F = 3.96). For PC2 (Session 2), there was also no effect of sex (P > 0.93, df=1, F = 0.007), an effect of context (P = 0.043, df=1.8, F = 3.30) and but no significant interaction between the two factors (P > 0.14, df=1.8, F = 1.99). For PC3 (Session 2), there was also no effect of sex (P > 0.74, df=1, F = 0.10) or context (P = > 0.49, df=1.2, F = 0.56) and no significant interaction between the two factors (P > 0.43, df=1.2, F = 0.71).


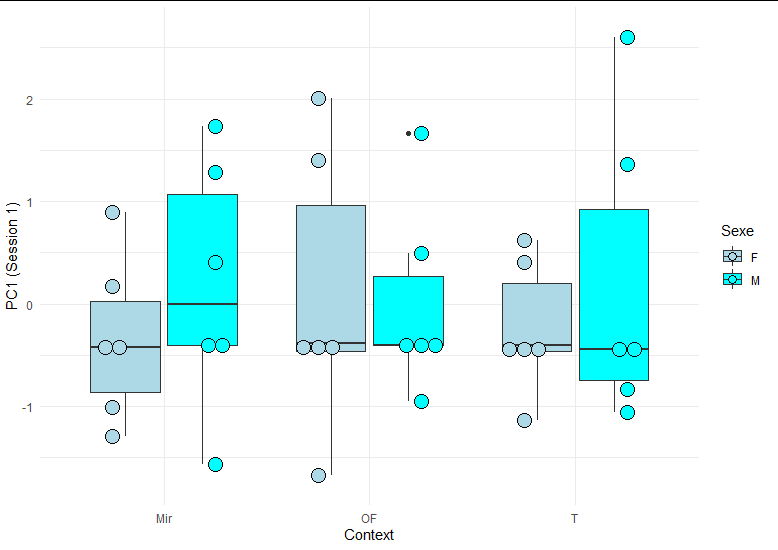


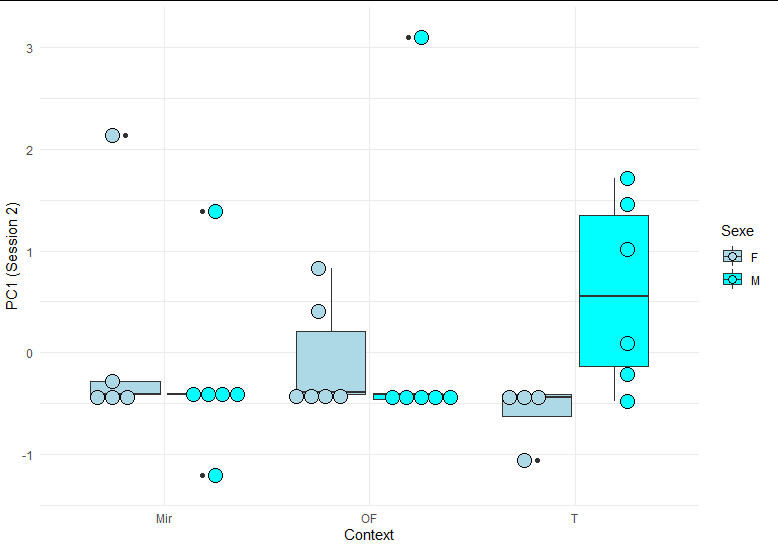


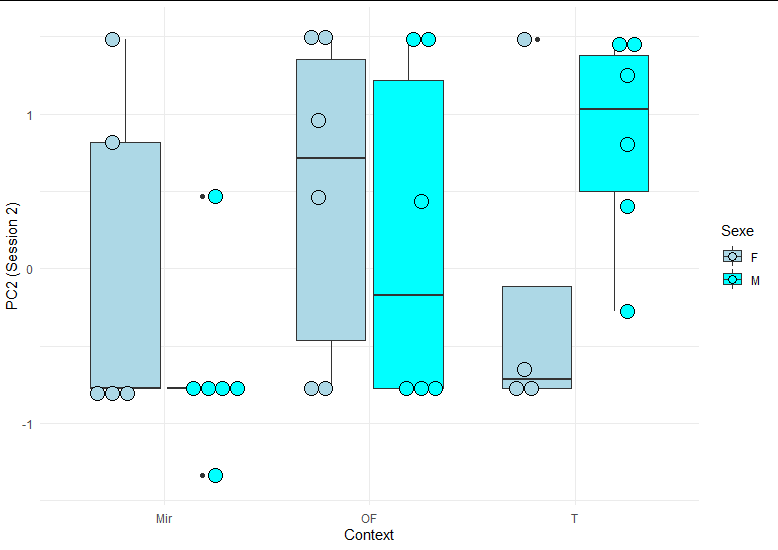


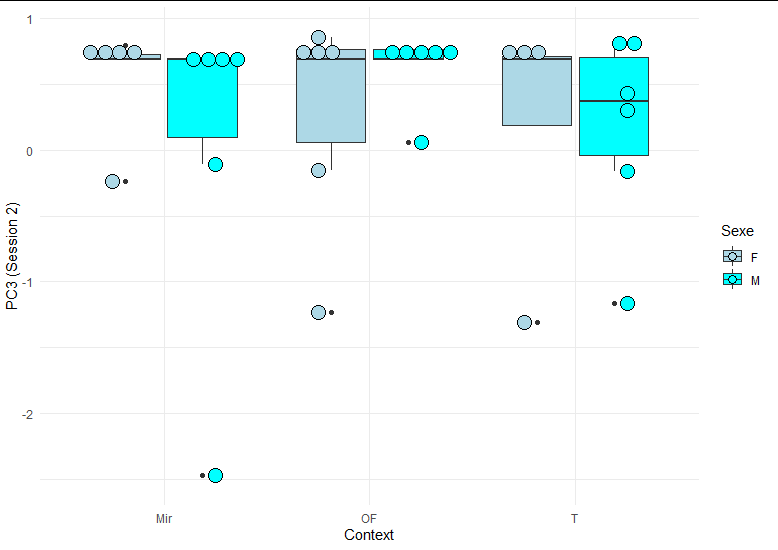

Supplement: Supplemental Information 2 [file peerj-09-11393-s002.docx]
